# Assessment of Long-Read Sequencing-Based Congenital Adrenal Hyperplasia Genotyping Assay for Newborns in Fujian, China

**DOI:** 10.3390/ijns11010022

**Published:** 2025-03-13

**Authors:** Xudong Wang, Xingxiu Lu, Faming Zheng, Kun Lin, Minjuan Liao, Yi Dong, Tiantian Chen, Ying He, Mei Lu, Jing Chen, Yanfang Li, Yulin Zhou

**Affiliations:** 1 Xiamen Newborn Screening Center, Department of Pediatrics, Women and Children’s Hospital, School of Medicine, Xiamen University, Xiamen 361102, China; xmxszx@163.com (Y.H.); 15960204233@163.com (Y.L.); 2 United Diagnostic and Research Center for Clinical Genetics, School of Public Health, Xiamen University, Xiamen 361100, China; luxingxiu@stu.xmu.edu.cn; 3 Center of Neonatal Disease Screening, Quanzhou Maternity and Children’s Hospital, Quanzhou 362000, China; 22192325@163.com; 4 Prenatal Diagnosis Center, The Affiliated Hospital of Putian University, Putian University, Putian 351100, China; linkun2017@126.com; 5 Longyan Newborn Screening Center, The First Hospital of Longyan City, Longyan 364099, China; LYXSFZX@163.com; 6 Screening Center of Neonatal Genetic Metabolic Disease, Ningde Maternity and Child Health Care Hospital, Ningde 352100, China; 7 Zhangzhou Newborn Screening Center, Zhangzhou Maternity and Child Care Hospital, Zhangzhou 363000, China; luehang@163.com; 8 Department of Pediatric Endocrinology, Women and Children’s Hospital, School of Medicine, Xiamen University, Xiamen 361102, China; lm800529@126.com; 9 Department of Pediatrics, Women and Children’s Hospital, School of Medicine, Xiamen University, Xiamen 361102, China; chenjing8469899@126.com; 10 Department of Child Health, Women and Children’s Hospital, School of Medicine, Xiamen University, Xiamen 361102, China

**Keywords:** congenital adrenal hyperplasia, long-read sequencing, newborn screening, second-tier screening, combined screening strategy

## Abstract

Long-read sequencing (LRS) provides comprehensive genetic information, but research of LRS applied to congenital adrenal hyperplasia (CAH) newborn screening is limited. This study aimed to evaluate the clinical utility of LRS in genetic diagnosis and second-tier newborn screening. Neonates born between January 2017 and December 2022 in Fujian, China, were recruited for biochemical and LRS-based genetic screening assay. The LRS covers the entire gene regions and exon–intron boundary regions for *CYP21A2*, *CYP11B1*, *CYP17A1*, *HSD3B2*, and *StAR*. In this retrospective study, 1,774,555 newborns received 17α-OHP screening, yielding a screening positive rate of 0.20%. Of these high-risk neonates, 3411 were successfully recalled for re-evaluation. Finally, 66 neonates were diagnosed with CAH, with a positive predictive value of 28.82%. Based on this data, the overall prevalence of CAH in Fujian was estimated to be 1:26,883. LRS was performed on 57 neonates with 21-hydroxylase deficiency (21-OHD) and 109 variant alleles were identified. The c.293-13C>G variant (31.19%) was the most prevalent in Fujian. Additionally, 647 neonates with suspected positive results were genotyped, and 41 were identified as carriers, with carrier frequencies of 1:18 for *CYP21A2*, 1:162 for *HSD3B2*, and 1:324 for *CYP17A1* in Xiamen. Therefore, LRS can provide comprehensive genotypes in approximately 1.5 days at a cost of less than $20 USD per sample, and effectively improve screening efficiency, reduce anxiety of parents during newborn screening (NBS), and shorten the time to referral of CAH patients (approximately 10 days). Such a combined screening strategy is worthy to be recommended for NBS programs in the future.

## 1. Introduction

Congenital adrenal hyperplasia (CAH, OMIM #201910) is a group of autosomal recessive inherited diseases, which is characterized by enzymatic deficiencies in adrenal cortical hormone synthesis [1]. Clinically, more than 90% of CAH cases are due to 21-hydroxylase deficiency (21-OHD), which leads to the impaired production of cortisol, aldosterone, and androgen. 21-OHD can be classified into three subtypes according to severity of enzymatic deficiency: classic salt-wasting (SW, early onset), classic simple virilizing (SV, early onset), and non-classic congenital adrenal hyperplasia (NCCAH) [2]. The incidence of classic CAH in China is approximately 1:23,024 live births, but NCCAH is much more prevalent, with an incidence of 1:1000 live births [3,4,5]. The carrier frequency of classic CAH allele is approximately 1:60 in the general population [6].

21-OHD is mainly caused by variants of the 21-hydroxylase (*CYP21A2*) gene, which is located at chromosome 6p21.3 and consists of 10 exons and 9 introns. A nonfunctional pseudogene, *CYP21A1P*, is located at a distance of 30 kb of *CYP21A2* gene [7]. The nucleotide sequences of the two genes share high homology, with 98% homology in exons and 96% in introns [8]. High homology and tandem repeats between the two genes increase the incidence of gene conversion, unequal crossovers, gene deletion, duplication, and formation of nonfunctional chimeric genes (*CYP21A1P*/*CYP21A2* or *TNXA*/*TNXB* chimeras) [9]. Therefore, approximately 75% of the variants (especially single nucleotide variations (SNVs) and small insertions and deletions) are derived from nonfunctional pseudogene *CYP21A1P* by micro-conversion, and the remaining 25% are large deletions or chimeric genes (*CYP21A1P*/*CYP21A2* chimeras or *TNXA*/*TNXB* chimeras) [10,11].

Newborn screening (NBS) is a successful and comprehensive public health program that can effectively identify infants at risk of rare childhood-onset disorders and administer timely treatment before the onset of said disorders. The current recommendation is to measure 17-hydroxyprogesterone (17α-OHP) level in newborn screening, with varied false-positive rates between 0.4% and 9.3% [12]. Newborn screening for 17α-OHP concentration can effectively identify the severe life-threatening SW and SV subtypes but may not identify the less severe SV and NC subtypes [13,14]. Thus, genotyping analysis is of great importance for confirmatory diagnosis, genetic counseling, and optimum administration (drug intervention and long-term follow-up) [15]. Multiplex ligation-dependent probe amplification (MLPA) and Sanger sequencing are the traditional genetic diagnostic methods used in most clinical laboratories. Both methods are regarded as the gold standard for *CYP21A2* genotyping, but they are labor-intensive and require an accurate experimental design. Recently, next-generation sequencing (NGS)-based genotyping assay targeting candidate genes has become the most efficient molecular diagnostic approach for genetic diseases; however, it is not suitable for *CYP21A2* genotyping because of the short sequencing reads and low analytical sensitivity in high homology sequences and intergenic conversion [16]. Long-read sequencing (LRS), a novel and robust approach with much longer sequencing reads, can directly analyze full-length genes and their flanking regions, identify the cis- and trans-configuration of multiple variants and discriminate highly homologous genes (such as *CYP21A2* and *CYP21A1P*) [12,17]. LRS-based CAH genotyping assay has been established and consists of 6 locus-specific long-range PCR (LR-PCR) fragments to cover the entire gene regions and exon–intron boundary regions for *CYP21A2*, *CYP11B1*, *CYP17A1*, *HSD3B2*, and *StAR* genes [17]. In the present study, LRS-based CAH genotyping was introduced into the NBS program to systematically evaluate its clinical utility in the genetic diagnosis of patients with CAH and second-tier newborn screening for CAH.

## 2. Materials and Methods

### 2.1. Study Design

**Figure 1:**
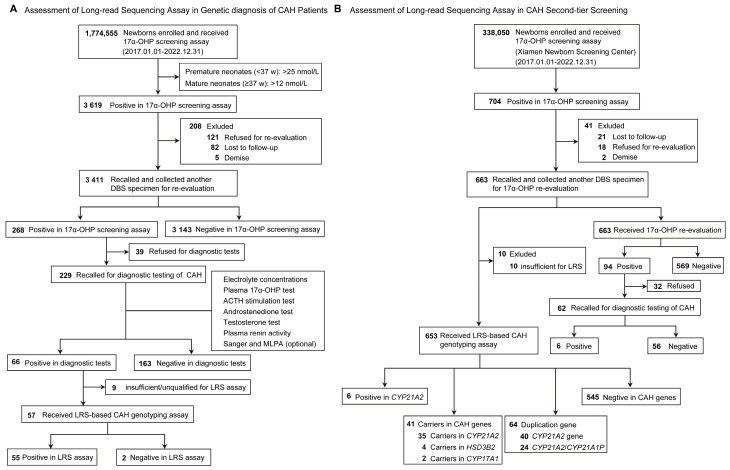
Assessment of long-read sequencing-based CAH genotyping assay for CAH newborn screening program: (**A**) assessment of long-read sequencing assay in genetic diagnosis of CAH patients; (**B**) assessment of long-read sequencing assay in CAH second-tier screening in Xiamen City.

This study was developed to assess the clinical utility of long-read sequencing of CAH genes (Figure 1), which included: (1) genetic diagnosis of patients with CAH in the cohort of neonates from six Newborn Screening Centers and (2) CAH second-tier newborn screening in the cohort of neonates from Xiamen Newborn Screening Center. This population-based retrospective cohort study was conducted at six Newborn Screening Centers: (1) Xiamen Newborn Screening Center, (2) Quanzhou Newborn Screening Center, (3) Longyan Newborn Screening Center, (4) Ningde Newborn Screening Center, (5) Zhangzhou Newborn Screening Center, and (6) Putian Newborn Screening Center. In this study, the inclusion criteria were as follows: (1) the neonates were born between January 2017 and December 2022 in Fujian; (2) the neonates received the 17α-OHP screening after 72 h of life; (3) the parents or their guardians agreed to have their babies undergo the genetic screening of CAH. The exclusion criteria were as follows: (1) the neonates’ dried blood spot (DBS) samples from the recalling for re-examination were insufficient/unqualified for the CAH genetic screening assay; (2) neonates were dead before recall for re-evaluation, refused to recall for re-evaluation, or lost to follow-up. In addition, these CAH patients were previously genotyped by MLPA analysis and Sanger sequencing. The study was approved by the Ethics Committees of Women and Children’s Hospital at the School of Medicine of Xiamen University (KY-2023-034-K02). All experiments were performed in accordance with the Declaration of Helsinki and informed consent was obtained from the patients or their guardians.

### 2.2. Newborn Screening for 17α-OHP Level

Neonatal heel blood specimens were collected after 72 h of life by well-trained nurses. The blood specimens were spotted on Whatman 903 filter papers (Guthrie card) and dried at room temperature (18–25 °C) while avoiding exposure to sunlight. DBS samples were then delivered to local newborn screening centers for 17α-OHP screening assay. In the study, 17α-OHP concentrations were measured using the GSP Neonatal 17α-OHP progesterone kit (Perkin Elmer, Turku, Finland) on a Genetic Screening Processor (GSP^®^) instrument (Wallac Oy, Turku, Finland). Based on the manufacturer’s recommendation, the neonate should be screening positive for CAH when the 17α-OHP concentration is >12 nmol/L for mature neonates (≥37 weeks), and >25 nmol/L for premature neonates (<37 weeks), respectively. Neonates with a positive value in the first screening assay were contacted and collected another DBS sample for re-evaluation using a GSP Neonatal 17α-OHP progesterone kit.

### 2.3. LRS-Based CAH Genotyping Assay

**Figure 2:**
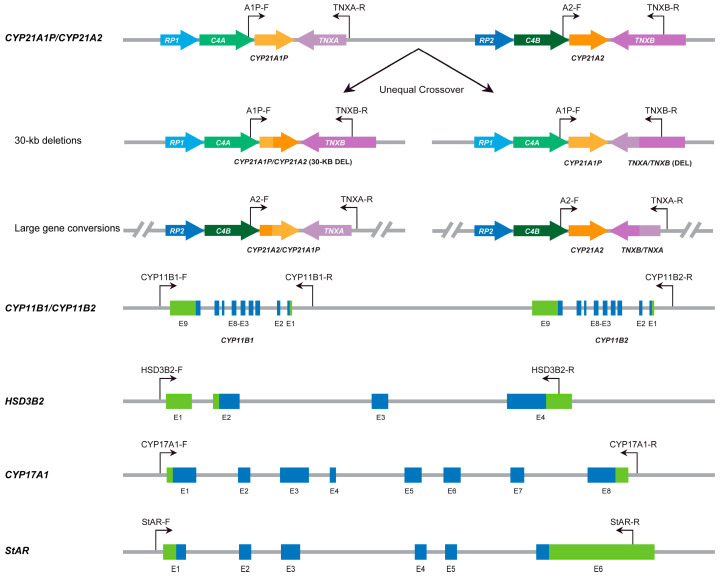
Positions of multiplex PCR-specific primer pairs targeting *CYP21A2*, *CYP11B1*, *CYP17A1*, *HSD3B2*, and *StAR* in LRS-based genotyping assay.

Genomic DNA was extracted from the DBS samples using a mini-nucleic acid extraction kit (Concert Bio, Xiamen, China) according to the manufacturer’s instructions. DNA concentration and quality were measured using a NanoDrop 2000 spectrophotometer (Thermo Fisher, Waltham, MA, USA). Long-read sequencing of CAH genes was performed at Berry Genomics Corporation (Being, China), and the protocol was as previously reported [17]. Briefly, genomic DNA was amplified using specific primers targeting the *CYP21A2*, *CYP11B1*, *CYP17A1*, *HSD3B2*, and *StAR* genes (Figure 2). Different amplicons were purified, end-repaired, and ligated with unique barcoded adaptors to build pre-sequencing single-molecule real-time (SMRT) libraries, which were further processed using Sequel Binding Kit 2.0 and Internal Control Kit 1.0 (Pacific Biosciences, California, USA) and sequenced on the Sequel II platform (Pacific Biosciences, California, USA). Raw data generated on the platform were processed using circular consensus sequencing (CCS) software version 6.0.0 (Pacific Biosciences, California, USA) to create CCS reads. These CCS reads were then demultiplexed and aligned to the reference genome hg38. Single nucleotide variations (SNVs) and small insertions and deletions (indels) were identified using FreeBayes 1.3.4. The *CYP21A2* and *CYP21A1P* genes, as well as the *CYP21A1P/CYP21A2* and *CYP21A2/CYP21A1P* chimeras, were identified using different primer pairs and two constructed references: *CYP21A2-TNXB* (chr6:32037179-32046548) and *CYP21A1P-TNXA* (chr6:32004843-32013648). The single nucleotide variations identified by LRS-based genotyping assay but not previously detected required confirmation by Sanger sequencing.

### 2.4. Statistical Analysis

All statistical analyses were performed using Excel 2021 and SPSS software version 22. The 95% CIs were calculated using *R* version 4.3.2 (R Foundation for Statistical Computing, Vienna, Austria).

## 3. Results

### 3.1. Study Population

**Figure 3:**
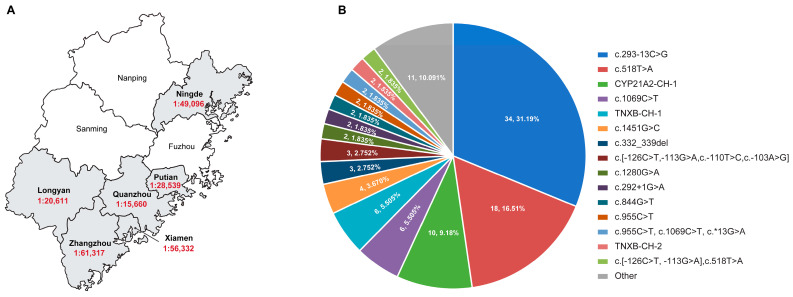
Incidence of CAH and Variants Distribution in Fujian Province: (**A**) incidence of CAH in 6 newborn screening centers from Fujian Province; (**B**) distribution of variants identified in 58 CAH patients from Fujian Province.

From January 2017 to December 2022, a total of 1,774,555 neonates were initially screened for CAH at six Newborn Screening Centers of Fujian Province (Figure 1A). In the 17α-OHP screening assay, 3619 infants were positive for the 17α-OHP level, yielding a screening positive rate of 0.20% (3619/1,774,555, 95% CI, 0.20% to 0.21%). Of these neonates with positive results in first-tier screening, 3411 were successfully recalled and another DBS specimen was collected for re-evaluation, with a recall rate of 94.25% (3411/3619, 95% CI, 93.4% to 95.0%). In the second screening, 268 infants remained positive in the second screening with a screening positive rate of 7.9% (268/3411, 95% CI, 6.98–8.81%). Afterwards, 229 infants were recalled for subsequent diagnostic tests. Ultimately, 66 neonates were diagnosed with CAH based on clinical phenotypes and genetic analyses (MLPA and Sanger sequencing), with a positive predictive value (PPV) of 28.82% (66/229, 95% CI, 23.05–35.15%). Based on these data, the overall prevalence of CAH in Fujian Province was estimated to be 1:26,883, with an incidence of 1:15,660 in Quanzhou City, 1:20,611 in Longyan City, 1:28,539 in Putian City, 1:49,096 in Ningde City, 1:61,317 in Zhangzhou City, and 1:56,332 in Xiamen City, respectively (Figure 3A).

### 3.2. LRS-Based CAH Genotyping Assay in CAH Genetic Diagnosis

**Figure 4:**
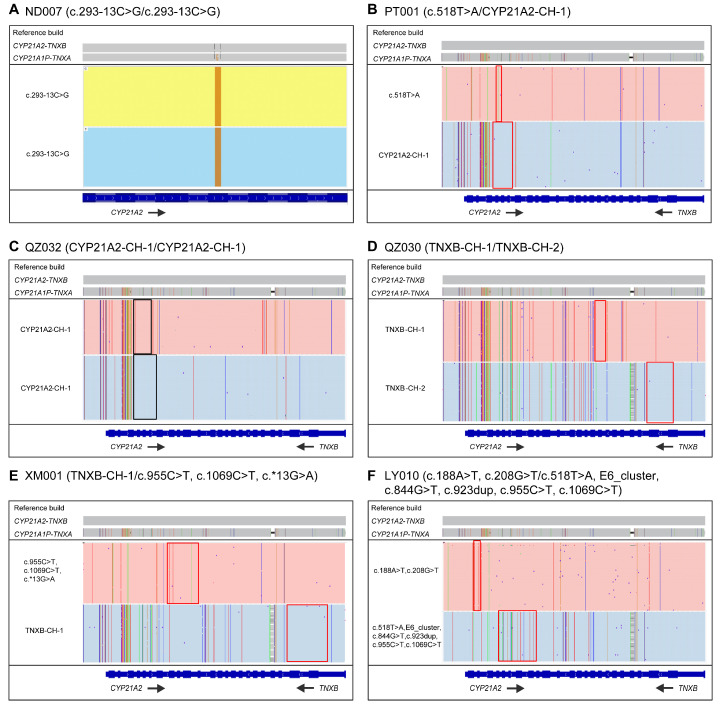
IGV plots of LRS results showing variations of CAH genes: (**A**) sample ND007 with *CYP21A2* SNV/indels; (**B**) sample PT001 with *CYP21A2* SNV and gene deletion; (**C**) sample QZ032 with homozygous *CYP21A2* deletions; (**D**) sample QZ030 with compound heterozygous *CYP21A2* deletions; (**E**) sample XM001 with *CYP21A2* SNVs and gene deletion; (**F**) sample LY010 with *CYP21A2* gene deletion and complex variations.

A total of 57 genomic DNA samples were extracted from DBS specimens and delivered to an independent laboratory (Berry Genomics, Beijing, China) for long-read sequencing-based CAH assay. These samples were genotyped previously using MLPA and Sanger sequencing assays, which detected biallelic variants in 53 neonates, monoallelic variants in 2 neonates (XM004 and LY003), and no variants in 2 neonates (ND006 and ZZ004). The variant detection rate was 94.74% (108/114) (Supplementary Table S1). In the LRS-based CAH genotyping assay, 54 samples were identified with *CYP21A2* mutations in both alleles and 1 sample with *CYP21A2* variant in one allele, with a variant detection rate of 95.61% (109/114) (Supplementary Table S1). Among the 55 cases with *CYP21A2* variants, 28 samples were homozygous or compound heterozygous for SNVs or indels, 17 samples were compound heterozygous for deletion and SNV/indels, 2 samples were homozygous for deletion, 7 samples were compound heterozygous for SNV/indels and complex variants, and 1 sample was a carrier for *CYP21A2* SNV. In summary, 76 alleles with SNVs/indels of 12 types, 21 alleles with large deletions of 6 types, and 12 alleles with complex variations were identified by the LRS-based genotyping assay (Table 1). In this study, the variant c.293-13C>G (31.19%, 34/109) is the most prevalent variation in Fujian, followed by c.518T>A (16.51%, 18/109), *CYP21A2*-CH-1 (9.17%, 10/109), c.1069C>T (5.50%, 6/109), and *TNXB*-CH-1 (5.50%, 6/109) (Figure 3B, Table 1). The IGV plots of the selected samples directly show the SNV/indels of *CYP21A2* and deletions in *CYP21A2* (Figure 4).

### 3.3. LRS-Based CAH Genotyping Assay in CAH Second-Tier Screening

In order to evaluate the clinical utility of LRS-based genotyping assay in second-tier newborn screening, a retrospective study was conducted at Xiamen Newborn Screening Center (Figure 1B). A total of 338,050 newborns born in Xiamen City (1 January 2017–31 December 2022) received a 17α-OHP screening assay, and 704 newborns were considered to be positive for 17α-OHP level with a screening positive rate of 0.21% (704/338,050, 95% CI, 0.19% to 0.22%). Of the neonates with positive results in first-tier screening, 663 were successfully recalled for re-evaluation. A total of 653 samples qualified for the LRS-based CAH genotyping assay, and the remaining 10 samples were excluded because of insufficient/unqualified genomic DNA. Among these 653 neonates, 6 cases were identified with 2 variants in *CYP21A2* gene, and 41 were identified with 1 variant (35 with *CYP21A2* variant, 4 with *HSD3B2* variant, and 2 with *CYP17A1* variant) (Table 2). The results demonstrated that the c.371C>T variant in *CYP21A2* gene is the most prevalent in Xiamen, with a frequency of 1:46. Meanwhile, 64 (9.9%) patients had duplications of *CYP21A2*, of which 40 (6.2%) were duplications of *CYP21A2* and 24 (3.7%) were duplications of *CYP21A2*/*CYP21A1P* chimeras.

## 4. Discussion

Congenital adrenal hyperplasia due to 21-hydroxylase deficiency is the most common subtype and mainly resulted from *CYP21A2* variants [9]. Newborn screening for 21-OHD facilitates early diagnosis and timely administration during the pre-symptomatic stage. Owing to the false positive results of biochemical testing, molecular screening is of great importance for the classification and treatment of CAH patients [16,17,18]. In this retrospective study, we aimed to evaluate clinical utility of the LRS-based CAH genotyping in the genetic diagnosis of patients with CAH and second-tier newborn screening for CAH.

In our study, the incidence of CAH in Fujian Province was about 1:26,887 live births, which is close to the incidence of 1:23,024 in China but lower than 1:15,136 in Fuzhou [5]. This discrepancy may be due to geographical differences. To date, more than 200 pathogenic variants in *CYP21A2* gene have been identified and recorded in the database [6,15,19]. In the present study, a total of 109 *CYP21A2* alleles were identified in 57 patients with CAH. The c.293-13C>G variant is most prevalent in Fujian Province, which is consistent with previous studies [17,18]. This indicates that *CYP21A2* is the major pathogenic gene in patients with CAH in Fujian Province. Meanwhile, it is worth noting that the c.725 T>C (p.L242P) variant is identified in patient XM004 and confirmed by Sanger sequencing. To our knowledge, the variant has not been previously reported and is absent in the HGMD, ClinVar, dbSNP, and gnomAD databases. Based on Mutation Taster (probability: 1.000), SIFT (score: 0.004), and Polyphen-2 (score: 1.000 for HumDiv model and 1.000 for HumVar model, respectively), it was predicted to be potentially damaging to the structure/function of the protein. Similarly, in the present study, this variant was not identified in 647 neonates from Xiamen. Eventually, the variant was interpreted as likely pathogenic according to the ACMG Standards and Guidelines (PS4 + PM2 + PP3 + PP4) [20].

To our knowledge, this retrospective study is the first to apply LRS-based genotyping assay to simulate the combined biochemical and genetic screening strategy and further validate its clinical utility in second-tier newborn screening. The data showed that there were no individuals identified with two *CYP21A2* variants or other genes in 647 newborns except for six patients with CAH, which indicates that the cut-off value applied in 17α-OHP screening leads to more false positive results and unnecessary recalling. In the Xiamen population, c.955C>T is a common variant in classic form variant, accounting for 50.0%. Similarly, c.371C>T, c.844G>T, and c.[-126C>T, -113G>A] are common non-classic form variants, accounting for 80.0%. The data showed that the carrier rate of classic variants of *CYP21A2* was approximately 1:65 in the Xiamen population, which is consistent with previous studies [21]. Interestingly, the variant c.371C>T was common in 647 newborns with positive results in primary screening (34.15%), with a carrier rate of 1:46, but its clinical phenotype remains controversial (classic SW CAH or n-classic CAH patients) [15,22,23]. In addition, a high frequency of duplication in *CYP21A2* gene (9.9%) were identified in 647 individuals, so the duplication of *CYP21A2* should be paid more attention when performing 21-OHD genetic diagnosis and genetic counseling. LRS-based genotyping assay used in our study can directly identify point variants, small deletions/insertions, large deletions/duplications, and gene conversions, which is not only suitable for the identification of complex structural variants, but also can accurately distinguish affected patients from carriers if *CYP21A2* duplication and SNV/indel are on the same chromosome [17]. Although LRS-based genotyping assay can simultaneously detect variants in *CYP21A2*, *CYP11B1*, *CYP17A1*, *HSD3B2*, and *StAR* genes, newborn screening for 17α-OHP is mainly used to identify 21-OHD caused by variants in *CYP21A2*. Other CAH subtypes caused by *CYP11B1*, *CYP17A1*, *HSD3B2*, and *StAR* are usually not identified by 17α-OHP screening assay. Therefore, it may achieve better screening effectiveness if LRS-based genotyping assay and biochemical screening assay can be simultaneously used as first-tier screening.

**Figure 5:**
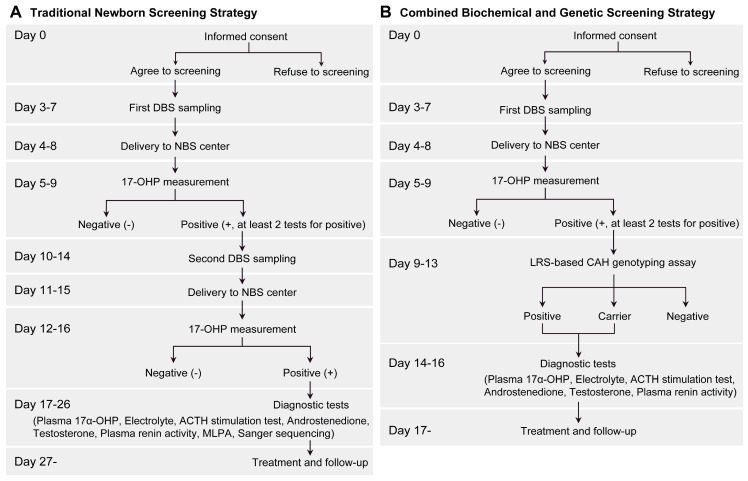
Comparison of different screening strategies in CAH newborn screening: (**A**) traditional newborn screening strategy in Xiamen City; (**B**) combined biochemical and genetic screening strategy. ACTH, adrenocorticotropic hormone; MLPA, multiplex ligation-dependent probe amplification; “at least 2 tests for positive”, at least 2 tests for positive in 17-OHP screening assay can be determined as screening positive.

Single-molecule, long-read sequencing has become a mainstream sequencing approach that targets single DNA molecules to perform real-time sequencing and produce much longer reads at a higher speed [24]. It has been successfully used in the diagnosis of cancers [25,26] and human genetic diseases [17,27,28,29,30]. Compared with the clinical performance and cost of MLPA and Sanger sequencing, LRS-based CAH genotyping is a promising method for 21-OHD diagnosis and second-tier newborn screening for CAH. First, it can provide comprehensive genetic information, directly analyze the copy number and cis–trans configuration of *CYP21A2* and *CYP21A1P*, and reveal the junction sites of chimeric genes without the need to recall other family members to further analysis compared to current approaches (MLPA and Sanger sequencing) [12,17]. It can provide a shorter turnaround time (approximately 1.5 days) to obtain genotype results, whereas MLPA and Sanger sequencing usually require approximately 2–3 days from sampling to reporting when these genotyping assays can be performed in our own laboratories. Second, incorporating genetic screening into neonatal 17α-OHP biochemical screening (Figure 5) can significantly reduce the unnecessary recalling and second DBS sampling, and shorten effectively the turnaround time from screening to diagnosis (26 days vs. 16 days). Meanwhile, non-classic CAH and carrier status can be identified, which cannot be detected by traditional biochemical screening and is of great significance for genetic counseling and further pregnancy. Third, clinical samples from different sources (especially DBS samples) could yield sufficient genomic DNA (>1 ng/μL) for LRS-based CAH genotyping assay. While MLPA and Sanger sequencing have a relatively low throughput and required a high-quality genomic DNA (>10 ng/μL), thus they are not suitable for large-scale newborn genetic screening. Fourth, LRS assay could simultaneously detect 384 samples in one assay, with the assistances of PacBio Sequel II platform and barcode system, at an overall cost of less than $20 USD per sample [17]. While the cost of MLPA assay for one sample is about $16.69 USD [31], it is well-known that MLPA cannot identify all pathogenic variants, requiring trio analysis and Sanger sequencing (about $50.07 USD), which further increases the cost. Consequently, LRS assay may have great potential in newborn screening for CAH (especially for the first-tier screening) if its cost can be reduced in the future.

However, this study had some limitations. First, our study is a retrospective study based on six NBS centers from Fujian Province, and the study of LRS-based genotyping assay as a second-tier screening assay was only conducted in Xiamen City. Therefore, the multicenter population-based prospective studies are required to systematically evaluate the incidence of CAH in Fujian and the clinical utility of long-read sequencing for newborn screening. Second, prospective studies are required to evaluate the cost-effectiveness of this combined genetic and biochemical screening strategy and promote its inclusion in NBS programs. Third, the functional impact of the c.725 T>C and c.371 C>T variants is unclear; therefore, the pathogenicity and clinical phenotype of the variants need to be confirmed in further studies.

## 5. Conclusions

In conclusion, LRS-based genotyping was a comprehensive method for detecting CAH-related genes and was applied to second-tier newborn screening and molecular diagnosis of CAH in newborns. The prevalence and variant spectrum of CAH in Fujian Province were demonstrated in detail, highlighting the importance of accurate classification, molecular diagnosis, and clinical intervention and management of patients with CAH. The findings of this study indicate that the integration of LRS-based genetic screening and traditional CAH biochemical screening will contribute to greatly reducing the rates of false-positives of biochemical screening and recall for re-evaluation, release the anxiety of parents during newborn screening, and further improve newborn screening efficiency. It can shorten the time to referral of CAH patients (approximately 10 days) without the need for a second heel puncture, while maintaining sensitivity of current NBS. However, a multicenter, population-based prospective study is needed to evaluate the clinical feasibility and cost-effectiveness of LRS in NBS programs.

## Figures and Tables

**Table 1:** Allele frequencies of *CYP21A2* variants in 57 CAH patients.

Variant Type	Nucleotide Change	Protein Change	Allele Number (*n* = 109)	Frequency (*n* = 109)
SNV/indels	c.293-13C>G	-	34	31.19%
	c.518T>A	p.Ile173Asn	18	16.51%
	c.1069C>T	p.Arg357Trp	6	5.50%
	c.1451G>C	p.Arg484Pro	4	3.67%
	c.332_339del	p.Gly111Valfs	3	2.75%
	c.1280G>A	p.Arg427His	2	1.83%
	c.292+1G>A	-	2	1.83%
	c.844G>T	p.Val282Leu	2	1.83%
	c.955C>T	p.Gln319Ter	2	1.83%
	c.1451_1452delinsC	p.Arg484fs	1	0.92%
	c.949C>T	p.Arg317Ter	1	0.92%
	c.725C>T	p.Leu242Pro	1	0.92%
Deletion	CYP21A2-CH-1	-	10	9.17%
	TNXB-CH-1	-	6	5.50%
	TNXB-CH-2	-	2	1.83%
	CYP21A2-CH-3	-	1	0.92%
	TNXB-CH-3	-	1	0.92%
	CYP21A2-CH4	-	1	0.92%
Complex mutations	c.[-126C>T, -113G>A, c.-110T>C, c.-103A>G]	-	3	2.75%
	c.955C>T, c.1069C>T, c.*13G>A	p.Gln319Ter, p.Arg357Trp,	2	1.83%
	c.[-126C>T, -113G>A], c.518T>A	p.Ile173Asn	2	1.83%
	c.[-126C>T, -113G>A]	-	1	0.92%
	c.[-126C>T, -113G>A, -110T>C, -103A>G], c.92C>T	p.Pro31Leu	1	0.92%
	c.[-113G>A, -110T>C]	-	1	0.92%
	c.188A>T, c.208G>T	p.His63Leu, p.Val70Leu	1	0.92%
	c.518T>A, E6_cluster (c.710T>A, c.713T>A, c.719T>A), c.844G>T, c.923dup, c.955C>T, c.1069C>T	p.Ile173Asn, p.Val282Leu, p.Leu308Phefs p.Gln319Ter, p.Arg357Trp	1	0.92%

Abbreviations: SNV, single nucleotide variation; Indels, small insertions and deletions.

**Table 2:** Variants identified in 647 suspicious positive neonates.

Gene	Variant Type	Genotype	Protein Change	Phenotype	Case Number	Carrier RATE
*CYP21A2*	SNV	c.955C>T/+	p.Gln319Ter	SW/+	5/647 (0.77%)	1:65
	SNV	c.293-13C>G/+	-	SW/+	2/647 (0.31%)	
	SNV	c.518T>A/+	p.Ile173Asn	SV/+	2/647 (0.31%)	
	Deletion	CYP21A2-CH-8/+	-	Classic CAH	1/647 (0.15%)	
	SNV	c.371C>T/+	p.Thr124Ile	NC/+	14/647 (2.16%)	1:26
	SNV	c.844G>T/+	p.Val282Leu	NC/+	3/647 (0.46%)	
	SNV	c.[-126C>T, -113G>A]/+	-	NC/+	3/647 (0.46%)	
	SNV	c.913G>A/+	p.Val305Met	NC/+	2/647 (0.31%)	
	SNV	c.1099C>T/+	p.Arg367Cys	NC/+	1/647 (0.15%)	
	SNV	c.-126C>T/+	-	NC/+	1/647 (0.15%)	
	SNV	c.143A>G/+	p.Tyr48Cys	NC/+	1/647 (0.15%)	
	Dup	*CYP21A2*	-		40/647 (6.18%)	1:10
	Dup	*CYP21A2*/*CYP21A1P*	-		24/647 (3.71%)	
*HSD3B2*	SNV	c.1003C>T/+	p.Arg335Ter		2/647 (0.31%)	1:162
	SNV	c.674T>A/+	p.Val225Ala		1/647 (0.15%)	
	SNV	c.244G>A/+	p.Ala82Thr		1/647 (0.15%)	
*CYP17A1*	SNV	c.715C>T/+	p.Arg239Ter		1/647 (0.15%)	1:324
	Indels	c.1459_1467del/+	p.Asp487_Phe489del		1/647 (0.15%)	

Abbreviations: SNV, single nucleotide variation; Indels, small insertions and deletions; Dup, duplication.

## Data Availability

The raw data can be obtained on request from the corresponding authors.

## Supplementary Materials

The following supporting information can be downloaded at: https://doi.org/10.17632/syp68bvdc6.1, Table S1, Detailed information of 57 patients with CAH from Fujian Province.
